# Optimal conspicuity of pancreatic ductal adenocarcinoma in virtual monochromatic imaging reconstructions on a photon-counting detector CT: comparison to conventional MDCT

**DOI:** 10.1007/s00261-023-04042-5

**Published:** 2023-10-05

**Authors:** Josua A. Decker, Judith Becker, Mark Härting, Bertram Jehs, Franka Risch, Luca Canalini, Claudia Wollny, Christian Scheurig-Muenkler, Thomas Kroencke, Florian Schwarz, Stefanie Bette

**Affiliations:** 1 Diagnostic and Interventional Radiology, Faculty of Medicine, University Hospital Augsburg, University of Augsburg, Stenglinstr. 2, 86156 Augsburg, Germany; 2 Centre for Advanced Analytics and Predictive Sciences (CAAPS), University of Augsburg, Universitätsstr. 2, 86159 Augsburg, Germany; 3 Medical Faculty, Ludwig Maximilian University Munich, Bavariaring 19, 80336 Munich, Germany; 4 Institute for Radiology, DONAUISAR Hospital Deggendorf-Dingolfing-Landau, Perlasberger Str. 41, 94469 Deggendorf, Germany

**Keywords:** Photon-counting detector computed tomography, Pancreatic ductal adenocarcinoma, Virtual monoenergetic imaging, Contrast-enhanced CT

## Abstract

**Purpose:**

To analyze the conspicuity of pancreatic ductal adenocarcinoma (PDAC) in virtual monoenergetic images (VMI) on a novel photon-counting detector CT (PCD-CT) in comparison to energy-integrating CT (EID-CT).

**Methods:**

Inclusion criteria comprised initial diagnosis of PDAC (reference standard: histopathological analysis) and standardized contrast-enhanced CT imaging either on an EID-CT or a PCD-CT. Patients were excluded due to different histopathological diagnosis or missing tumor delineation on CT. On the PCD-CT, 40–190 keV VMI reconstructions were generated. Image noise, tumor-to-pancreas ratio (TPR) and contrast-to-noise ratio (CNR) were analyzed by ROI-based measurements in arterial and portal venous contrast phase. Two board-certified radiologist evaluated image quality and tumor delineation at both, EID-CT and PCD-CT (40 and 70 keV).

**Results:**

Thirty-eight patients (mean age 70.4 years ± 10.3 [range 45–91], 27 males; PCD-CT: n=19, EID-CT: n=19) were retrospectively included. On the PCD-CT, tumor conspicuity (reflected by low TPR and high CNR) was significantly improved at low-energy VMI series (≤ 70 keV compared to > 70 keV), both in arterial and in portal venous contrast phase (P < 0.001), reaching the maximum at 40 keV. Comparison between PCD-CT and EID-CT showed significantly higher CNR on the PCD-CT in portal venous contrast phase at < 70 keV (P < 0.016). On the PCD-CT, tumor conspicuity was improved in portal venous contrast phase compared to arterial contrast phase especially at the lower end of the VMI spectrum (≤ 70 keV). Qualitative analysis revealed that tumor delineation is improved in 40 keV reconstructions compared to 70 keV reconstructions on a PCD-CT.

**Conclusion:**

PCD-CT VMI reconstructions (≤ 70 keV) showed significantly improved conspicuity of PDAC in quantitative and qualitative analysis in both, arterial and portal venous contrast phase, compared to EID-CT, which may be important for early detection of tumor tissue in clinical routine. Tumor delineation was superior in portal venous contrast phase compared to arterial contrast phase.

**Supplementary Information:**

The online version contains supplementary material available at 10.1007/s00261-023-04042-5.

## Introduction

Pancreatic ductal adenocarcinoma (PDAC) remains a leading cause of cancer death; despite advances in diagnosis and treatment in recent years, the 5-year survival rate is low, ranging from 3 to 15% [1–5]. Many patients with PDAC present with symptoms such as jaundice or abdominal pain. In most cases, the tumor is detected at a late stage, resulting in unresectable tumor [6]. Therefore, early detection of the tumor is a major goal to improve patient survival.

Many imaging modalities are available for the diagnosis of PDAC and have been evaluated in recent years [6]. Endoscopic ultrasound is the most sensitive method to detect PDAC [7]; however, it is an invasive method and is often used as an adjunct in clinical routine [6].

Multi-detector computed tomography (MDCT) is the gold standard in imaging of the pancreas [6]. It has a high availability and shows a sensitivity of 76–92% and a specificity of 67% for the detection of PDAC [6, 8–10]. Multiphase CT is the standard imaging technique and has a high sensitivity for detection of PDAC and hepatic metastases [11–13]. In the pancreatic phase, performed after a delay of about 35–45 s after contrast administration, the tumor can be delineated as a hypoattenuating mass. The portal venous phase (75 s after contrast injection) is important for delineation of metastases, whereas the PDAC itself often shows comparable CT-values to pancreatic tissue [11].

Virtual monoenergetic images (VMI) derived from dual-energy CT (DECT) show promising results for improving abdominal CT imaging, e.g., the better conspicuity of liver metastases at lower keV levels [14, 15]. The reconstruction of VMI can be performed after image acquisition and is based on the ability of DECT to perform material decomposition. Low keV reconstruction (minimum 40 keV) as well as high keV reconstructions can be carried out. However, low keV reconstructions also show higher image noise which might limit the applicability in clinical routine [16] and could be overcome with noise-optimized VMI (VMI+) [17]. At lower keV-levels, the iodine signal is maximized, resulting in a better contrast, but also in higher image noise on DECT [18]. Previous studies showed the optimized conspicuity of PDAC in low keV reconstructions using DECT, reaching a maximum at 40 keV [16, 19–23].

Recently, photon-counting detector CT (PCD-CT) has been introduced into clinical routine. These detectors are—in contrast to conventional energy-integrating detectors (EID-CT)—capable of directly converting x-ray photons into an electrical signal [24, 25]. The advantages of this new technology are reduced radiation dose, no electronic noise, improved spatial resolution and intrinsic spectral sensitivity in each scan [24–26]. These advantages, combined with the known improvement in lesion conspicuity at lower keV levels in DECT, may be a promising tool for the early detection of PDAC. Recent studies showed the improved conspicuity of liver metastases [15] as well as improved subjective image quality for abdominal imaging using low keV reconstructions on a PCD-CT [27].

Therefore, aim of this study was to analyze the potential of PCD-CT in the conspicuity of PDAC at different keV VMI reconstructions and different contrast phases in comparison to EID-CT.

## Materials and methods

This retrospective single center study was approved by the local ethics committee and the need to obtain informed consent was waived. The local database was queried for patients with the diagnosis of pancreatic cancer who had undergone contrast-enhanced CT of the (upper) abdomen on a novel Dual-Source Photon Counting CT Scanner (NAEOTOM Alpha, Siemens Healthineers, Erlangen, Germany) as part of routine clinical care between 04/2021 and 07/2021.

The local database was further queried for patients with the diagnosis of pancreatic cancer who had undergone contrast-enhanced CT of the (upper) abdomen on an energy-integrating-detector CT (20-slice MDCT Somatom AS20, Siemens Healthineers, Erlangen, Germany).

Patients’ medical charts were reviewed for the following parameters: age, gender, body mass index (kg/m^2^), tumor pathology and location and CTDI (mGy). Reference standard for the diagnosis of PDAC was histopathological analysis.

Inclusion criteria comprised age ≥ 18 years, contrast-enhanced CT of the abdomen either on a PCD-CT or and EID-CT between 04 and 07/2021, histopathological diagnosis of PDAC or in cases with missing histopathological diagnosis clear imaging findings consistent with PDAC. Exclusion criteria comprised other histopathological diagnosis and missing tumor delineation in CT.

### Imaging protocol

PCD-CT scans were performed on a dual-source photon-counting detector CT (NAEOTOM Alpha, Siemens Healthineers) as routine clinical acquisitions using a biphasic contrast injection protocol. A contrast bolus of 120 ml (Ultravist 300 mgI/mL, Bayer, Leverkusen, Germany) injected via an antecubital vein was followed by a saline bolus of 30 ml, both using a flow rate of 4.0 ml/s. The scan was bolus-triggered within the ascending aorta (after an attenuation of ≥ 120 HU) with a delay of 20 s for arterial contrast phase and 75 s for portal venous contrast phase.

All patients were scanned craniocaudally in a supine position from the diaphragm or upper thoracic aperture to the pelvis or the symphysis during a single breath-hold. For each scan, we applied the following parameters: acquisition mode with readout of spectral information (QuantumPlus, Siemens Healthineers), 120 kV tube voltage, 0.5 s rot. time, 144 × 0.4 mm collimation. Spectral series were generated using a soft-tissue kernel specifically developed for the spectral postprocessing of PCD-CT datasets (Qr40, QIR 3, Siemens Healthineers) and an enhanced DICOM file format containing spectral information (SPP, spectral postprocessing). Slice thickness was 1.0 mm with an increment of 1.0 mm.

In the comparison group all patients were scanned on an EID-CT (Somatom AS20, Siemens Healthineers) using the following acquisition settings: 120 kV tube voltage, 0.5 s rot. time, 144 × 0.4 mm collimation. On the EID-CT, identical settings for contrast application and delay for image acquisition were used.

### Image reconstruction and analysis

Using a dedicated workstation (Syngo.via VB60A, Siemens Healthineers) we performed post-processing of spectral data. For each patient and for each available contrast phase (arterial phase, portal venous phase), we generated VMI reconstructions (slice thickness of 1 mm, increment of 1mm and identical z-axis orientation) at the following keV levels: 40–80 keV in 5 keV increments and 80–190 keV in 10 keV increments. Post-processing of EID-CT images was also performed using a dedicated workstation (Syngo.via VB60A). Reconstructions were performed with identical slice thickness of 1 mm and an increment of 1 mm.

**Fig. 1:**
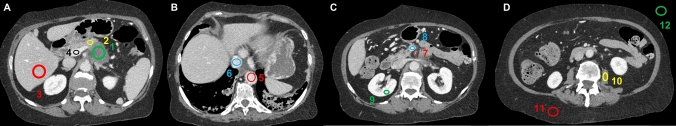
Image analysis and ROI-based measurement of mean CT-values (and standard deviation) in dedicated regions, shown at different slices in a contrast-enhanced CT in portal venous phase (**A**–**D**): (1) pancreatic tumor tissue, (2) pancreas, (3) liver parenchyma, (4) portal vein, (5) aorta, (6) inferior vena cava, (7) superior mesenteric artery, (8) superior mesenteric vein, (9) right renal cortex, (10) left psoas muscle, (11) subcutaneous tissue, and (12) air. ROI: region of interest

Further image analysis was performed using the open-source software *Fiji* [28], an image processing package based on *Image J*. For each patient and each available contrast phase, Regions of Interest (ROI) were manually placed in the following dedicated anatomic regions on the 40 keV dataset and automatically copied to all other VMI-datasets; ROI’s were placed with the maximum size to correctly describe the lesion/area: pancreatic tumor tissue (3 ROI’s), pancreas (tumor-adjacent pancreatic tissue, 3 ROI’s), liver tissue (3 ROI’s), aorta, portal vein, inferior vena cava, superior mesenteric artery, superior mesenteric vein, renal cortex (right side), psoas muscle (left side), subcutaneous tissue (right and left side), air (3 ROI’s). In total, 21 ROIs were measured at each keV level and at each contrast phase for each patient (Fig. 1).

All ROIs were positioned by a radiologist with 8 years of CT experience. From all ROI’s we assessed mean Hounsfield Units (HU) and standard deviation (SD). We calculated median image noise as median of all SD’s of all measured ROI’s at each keV level/contrast phase and as median of all ROI’s measured in subcutaneous tissue.

Tumor-to-pancreas contrast was calculated as ratio between CT-values measured in tumor and in pancreatic parenchyma:

$$TPR = \frac{{ Mean\,HU_{tumor} }}{{Mean\,HU_{pancreas} }}.$$

This method was performed analogous to the established tumor-to-liver ratio [15, 29]. Ratios were calculated between all three ROIs per region, resulting in nine tumor-to-pancreas ratios per keV level/contrast phase. Contrast-to-noise ratio (CNR) was calculated as described before:

$$CNR = \frac{{Mean\,HU_{pancreas} - Mean\,HU_{tumor} }}{{SD\,HU_{subcutaneous\,tissue} }}.$$

### Qualitative analysis

To assess subjective image quality, two board-certified radiologists reviewed all cases independently; in each case—if available—arterial and portal venous phase, on the PCD-CT 40 and 70 keV reconstructions. Overall image quality as well as tumor delineation were analyzed using a 5-point Likert scale ranging from 1 = poor to 5 = excellent.

### Statistical analysis

Analysis of descriptive data and statistical analyses were performed using SPSS 28.0 (IBM Corp. Released 2021. IBM SPSS Statistics for Windows, Version 28.0. Armonk, NY: IBM Corp). Data (CTDI, BMI, noise, CNR, and tumor-to-pancreas ratio) are non-normally distributed (shown by Kolmorogov-Smirnov-tests) and therefore presented as median and interquartile range (IQR). Mann-Whitney-U tests were performed to compare different groups. Bonferroni correction was performed for multiple testing. Statistically significant differences were assumed at P values ≤ 0.05.

## Results

### Patient population

On PCD-CT, 43 patients with a diagnosis of pancreatic cancer were identified from patients with available abdominal CT scan between 04/2021 and 07/2021. Seven patients were excluded due to missing delineation of tumor tissue on the CT scan, six patients were excluded due to previous Whipple procedure, eleven patients were excluded due to different histology after tumor resection (e.g., metastasis, benign tumor), resulting in 19 patients that were included in the study.

On the EID-CT, we identified 21 patients with pancreatic cancer and contrast-enhanced CT scan of the abdomen. Two patients were excluded due to different histology after resection/biopsy, resulting in 19 patients that were included in the study.

A total of 38 patients were included. Mean age was 68.7 ± 10.6 years (range 46–85) in the PCD-CT cohort and 72.1 ± 9.9 years (range 45–91) in the EID-CT cohort (P = 0.353). Median BMI was similar in both groups (PCD-CT: 22.50 kg/m^2^ [IQR: 20.81; 25.71], EID-CT: 22.50 kg/m^2^ [20.57; 26.71], P = 0.751). Also, CTDI-values were similar in both cohorts (PCD-CT: 6.68 mGy [5.59; 8.12], EID-CT: 7.64 mGy [5.33–13.05], P = 0.246) (Table 1).

**Table 1:** Patient characteristics

	PCD-CT	EID-CT	P value
Age (years)	68.7 ± 10.6 (46–85)	72.1 ± 9.9 (45–91)	0.353
Sex (male)	12/19	15/19	*0.042*
BMI (kg/m^2^)	22.50 (20.81–25.71)	22.50 (20.57–26.71)	0.751
CTDI (mGy)	6.68 (5.59–8.12)	7.64 (5.33–13.05)	0.246
Contrast phase			
Arterial and venous phase	10/19	10/19	1.000
Venous phase	9/19	9/19	
Tumor location			
Head, uncinate process	10/19	7/19	0.648
Body/tail	8/19	10/19	
Tumor tissue surrounding abdominal vessels	1/19	2/19	

Normally distributed data shown as mean ± SD (range), non-normally distributed data shown as median (interquartile range)

In 33/38 patients, pancreatic ductal adenocarcinoma was histologically confirmed either by surgery or biopsy. The remaining five patients had imaging features typical of PDAC (in arterial phase in contrast to pancreatic tissue hypoattenuating mass with lower/comparable CT-values in portal venous phase) [11]; histologic confirmation was not possible due to further treatment at other hospitals (n = 3) and inability to perform biopsy due to poor general health condition or anatomic conditions (n = 2).

### Image noise

**Fig. 2:**
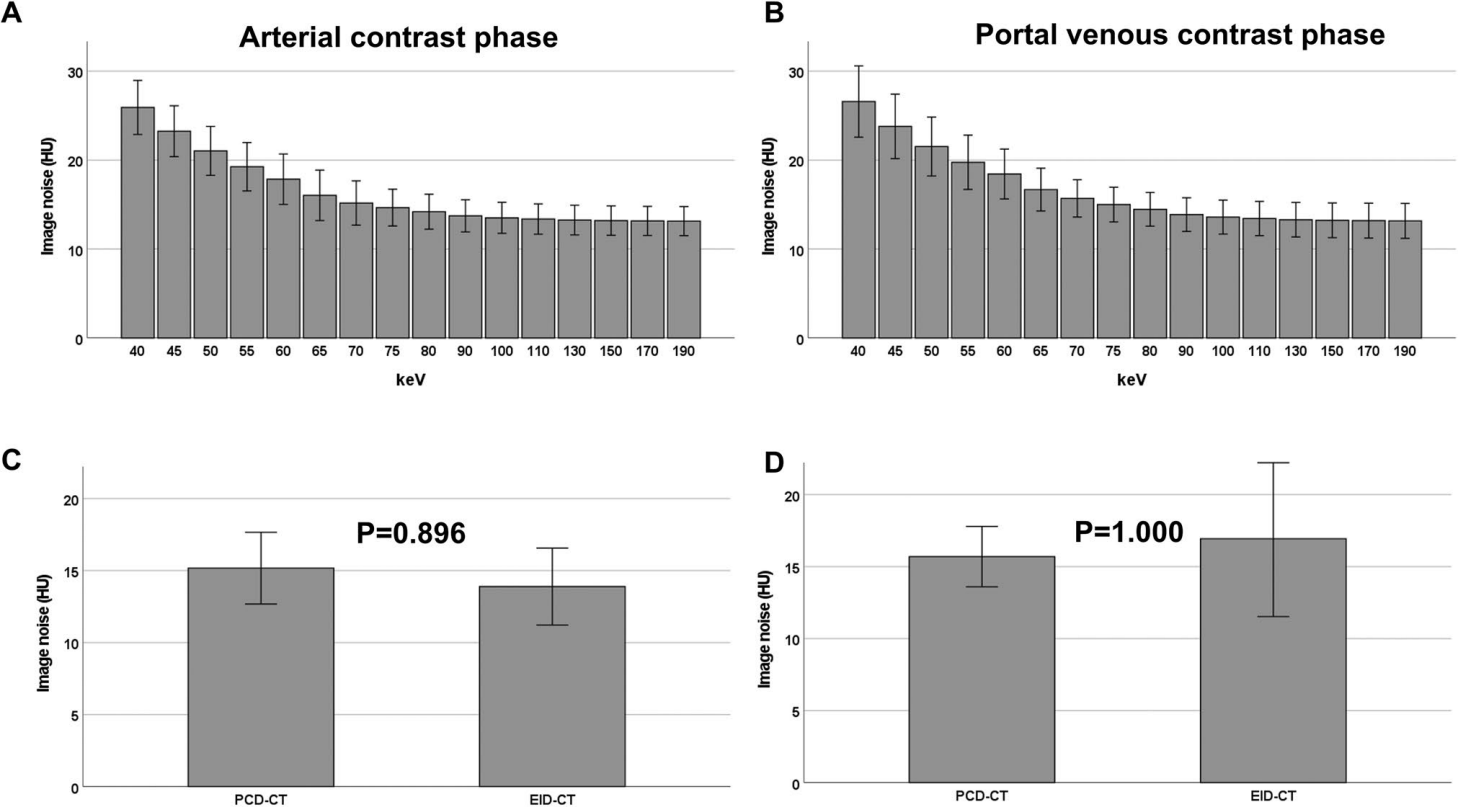
Median image noise (measured in subcutaneous tissue) on the PCD-CT in arterial (**A**) and portal venous contrast phase (**B**). Comparison between image noise on the PCD-CT (70 keV) and the EID-CT in arterial (**C**) and in portal venous contrast phase (**D**). ± 1 standard deviation. *PCD-CT* photon-counting detector CT, *EID-CT* energy-integrating detector CT

On the PCD-CT, image noise substantially decreased from a maximum at 40 keV in the arterial contrast phase (26.4 HU [24.8; 27.6]) to a median of 13.9 HU [13.4; 15.0] at 90 keV but, did not improve further at higher keV settings (Table 2, Fig. 2). Image noise (measured in subcutaneous tissue) was significantly higher at lower keV-levels (≤ 60 keV) on the PCD-CT compared to the EID-CT, both in arterial and in portal venous contrast phases. At 70 keV on the PCD-CT, image noise was comparable to the EID-CT in arterial phase (15.1 HU [14.1; 16.8] vs. 13.2 HU [12.0; 16.3], P = 0.896), and in portal venous phase (15.5 HU [14.3; 17.0] vs. 16.3 HU [13.0; 20.0], P = 1.000) (Table 2). Similar results were shown for image noise measured in all ROI’s (Supplementary Table 1).

**Table 2:** Median image noise at different keV levels and contrast phases

keV	Arterial phase		P value	Portal venous phase		P value
	PCD-CT	EID-CT		PCD-CT	EID-CT	
40	26.4 (24.8–27.6)	13.2 (12.0–16.3)	*0.016*	26.0 (23.7–29.7)	16.3 (13.0–20.0)	*0.016*
45	23.7 (22.0–24.9)		*0.016*	23.3 (21.0–26.4)		*0.016*
50	21.4 (20.0–23.0)		*0.016*	21.1 (18.8–23.7)		*0.016*
55	19.4 (18.0–21.5)		*0.016*	19.6 (17.3–22.0)		*0.032*
60	18.5 (16.3–20.0)		*0.016*	18.3 (16.2–20.1)		0.512
65	15.9 (14.5–17.8)		0.224	16.5 (15.2–18.2)		1.000
70	15.1 (14.1–16.8)		0.896	15.5 (14.3–17.0)		1.000
75	14.6 (14.0–16.1)		1.000	15.2 (13.5–15.9)		1.000
80	14.2 (13.7–15.6)		1.000	14.4 (12.6–15.4)		0.832
90	13.9 (13.4–15.0)		1.000	13.5 (12.3–15.2)		0.112
100	13.8 (13.1–14.6)		1.000	13.2 (12.2–14.9)		*0.048*
110	13.5 (12.9–14.5)		1.000	13.0 (12.1–14.7)		*0.016*
130	13.4 (12.6–14.3)		1.000	12.8 (11.8–14.5)		*0.016*
150	13.3 (12.5–14.3)		1.000	12.7 (11.8–14.3)		*0.016*
170	13.3 (12.4–14.2)		1.000	12.7 (11.8–14.2)		*0.016*
190	13.2 (12.4–14.2)		1.000	12.7 (11.7–14.2)		*0.016*

Image noise measured in subcutaneous fat. Data shown as median (interquartile range), P value shown after Bonferroni-correction

P value ≤ 0.05 shown in italics

### Tumor conspicuity

#### TPR

TPR is a measure for delineation of tumor tissue and calculated by the ratio between CT values in tumor tissue and in adjacent pancreatic tissue.

**Fig. 3:**
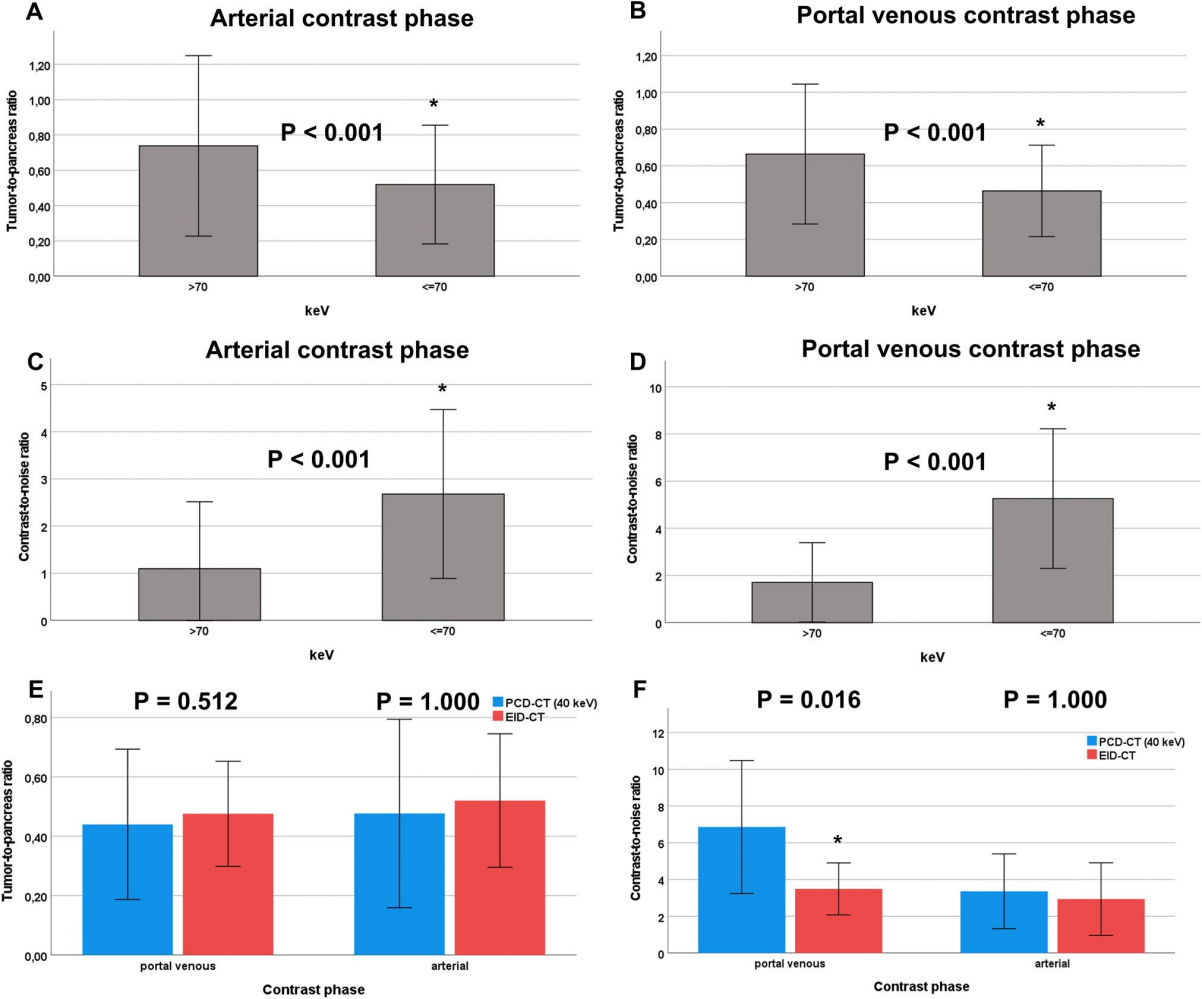
Tumor-to-pancreas ratio in arterial (**A**) and portal venous contrast phase (**B**) on the PCD-CT. Contrast-to-noise ratio in arterial (**C**) and portal venous contrast phase (**D**) on the PCD-CT. TPR (**E**) and CNR (**F**) compared between PCD-CT and EID-CT. ± 1 standard deviation. *P value ≤ 0.05. *TPR* tumor-to-pancreas ratio, *PCD-CT* photon-counting detector CT, *EID-CT* energy-integrating detector CT

Best conspicuity of pancreatic tumor tissue was shown in portal venous phase at the low end of VMI spectrum at 40 keV (TPR = 0.37 [0.20; 0.62]), increasing steadily to a maximum of 0.85 (0.48; 1.05) at 190 keV. TPR was significantly lower for VMI’s ≤ 70 keV compared to VMI’s > 70 keV, both in portal venous contrast phase (0.44 [0.23; 0.67] vs. 0.70 [0.37; 0.87], P < 0.001) and in arterial contrast phase (0.48 [0.30; 0.83] vs. 0.73 [0.36; 1.05], P < 0.001) (Fig. 3).

At lower keV-levels in arterial phase, no significant better tumor conspicuity was observed on the PCD-CT compared to the EID-CT (Table 3).

**Table 3:** Tumor-to-pancreas ratio and contrast-to-noise ratio at different keV levels and contrast phases on a PCD-CT and on an EID-CT

keV	Arterial phase			Portal venous phase		
	PCD-CT	EID-CT	P value	PCD-CT	EID-CT	P value
Tumor-to-pancreas ratio						
40	0.40 (0.26 to 0.79)	0.49 (0.37 to 0.69)	1.000	0.37 (0.20 to 0.62)	0.49 (0.32 to 0.62)	0.512
45	0.41 (0.27 to 0.82)		1.000	0.39 (0.21 to 0.65)		1.000
50	0.43 (0.28 to 0.85)		1.000	0.40 (0.22 to 0.65)		1.000
55	0.46 (0.29 to 0.87)		1.000	0.44 (0.23 to 0.65)		1.000
60	0.49 (0.30 to 0.88)		1.000	0.47 (0.25 to 0.65)		1.000
65	0.53 (0.30 to 0.86)		1.000	0.50 (0.26 to 0.66)		1.000
70	0.56 (0.32 to 0.85)		1.000	0.52 (0.28 to 0.68)		1.000
75	0.60 (0.34 to 0.89)		1.000	0.54 (0.30 to 0.71)		1.000
80	0.63 (0.35 to 0.84)		1.000	0.56 (0.30 to 0.73)		0.304
90	0.70 (0.37 to 0.90)		0.496	0.60 (0.30 to 0.78)		*0.016*
100	0.74 (0.38 to 0.95)		0.112	0.65 (0.34 to 0.81)		*0.016*
110	0.77 (0.38 to 1.00)		*0.032*	0.70 (0.36 to 0.84)		*0.016*
130	0.80 (0.40 to 1.09)		*0.016*	0.73 (0.41 to 0.92)		*0.016*
150	0.81 (0.40 to 1.12)		*0.016*	0.77 (0.44 to 0.98)		*0.016*
170	0.83 (0.40 to 1.15)		*0.016*	0.82 (0.46 to 1.01)		*0.016*
190	0.84 (0.41 to 1.19)		*0.016*	0.85 (0.48 to 1.05)		*0.016*
Contrast to to to noise ratio						
40	4.10 (0.94 to 4.63)	2.53 (1.59 to 4.49)	1.000	6.61 (4.39 to 9.51)	3.53 (2.45 to 4.33)	*0.016*
45	3.74 (0.77 to 4.19)		1.000	6.17 (3.90 to 8.64)		*0.016*
50	3.41 (0.67 to 3.93)		1.000	5.62 (3.47 to 7.84)		*0.016*
55	3.12 (0.62 to 3.76)		1.000	5.11 (3.08 to 7.13)		*0.016*
60	2.89 (0.50 to 3.58)		1.000	4.43 (2.80 to 6.47)		*0.016*
65	2.73 (0.44 to 3.67)		1.000	4.17 (2.70 to 5.95)		*0.016*
70	2.46 (0.44 to 3.36)		0.640	3.70 (2.39 to 5.31)		0.768
75	2.20 (0.39 to 3.05)		*0.032*	3.28 (2.06 to 4.73)		1.000
80	1.91 (0.87 to 2.75)		*0.016*	2.87 (1.82 to 4.22)		0.368
90	1.32 (0.49 to 2.32)		*0.016*	2.08 (1.44 to 3.31)		*0.016*
100	1.14 (0.23 to 2.09)		*0.016*	1.65 (1.14 to 2.69)		*0.016*
110	1.06 (0.00 to 1.91)		*0.016*	1.39 (0.82 to 2.23)		*0.016*
130	0.83 (− 0.28 to 1.72)		*0.016*	1.00 (0.37 to 1.74)		*0.016*
150	0.66 (− 0.39 to 1.61)		*0.016*	0.69 (0.08 to 1.45)		*0.016*
170	0.61 (− 0.49 to 1.53)		*0.016*	0.52 (− 0.05 to 1.28)		*0.016*
190	0.57 (− 0.55 to 1.48)		*0.016*	0.40 (− 0.15 to 1.21)		*0.016*

Data shown as median (interquartile range), P value shown after Bonferroni-correction

P value < 0.05 shown in italics

Comparison of VMI’s at 70 keV on the PCD-CT (which most closely resembles a standard polychromatic 120 kVp acquisition on the EID-CT) with reconstructions on the EID-CT did not show significantly better tumor conspicuity (reflected by TPR) on the PCD-CT, neither in arterial phase nor in portal venous phase (Table 2).

#### CNR

Besides TPR, CNR is a measure for tumor delineation in relation to image noise. Conspicuity of pancreatic tumor tissue (as reflected by CNR) was significantly improved at the lower end of the VMI spectrum in the portal venous phase (keV ≤ 65) compared to EID-CT reaching a maximum at 40 keV VMI reconstructions (40 keV PCD-CT: 6.61 [4.39; 9.51] vs. EID-CT: 3.53 [2.45; 4.33], P = 0.016). These differences remained significant up to 65 keV on the PCD-CT, (65 keV PCD-CT: 4.17 [2.70–5.95] vs. EID-CT: 3.53 [2.45; 4.33], P = 0.016).

In the arterial contrast phase, PCD-CT did not show significantly better tumor conspicuity (reflected by CNR) compared to EID-CT (Table 3, Fig. 3). On the PCD-CT, CNR was significantly higher at lower VMI’s ≤ 70 keV compared to higher VMI’s > 70, both in portal venous contrast phase (4.89 [3.08; 7.07] vs. 1.45 [0.48; 2.73], P < 0.001) and in arterial contrast phase (3.19 [0.70; 3.94] vs. 1.09 [− 0.12; 2.17], P < 0.001) (Fig. 3).

**Fig. 4:**
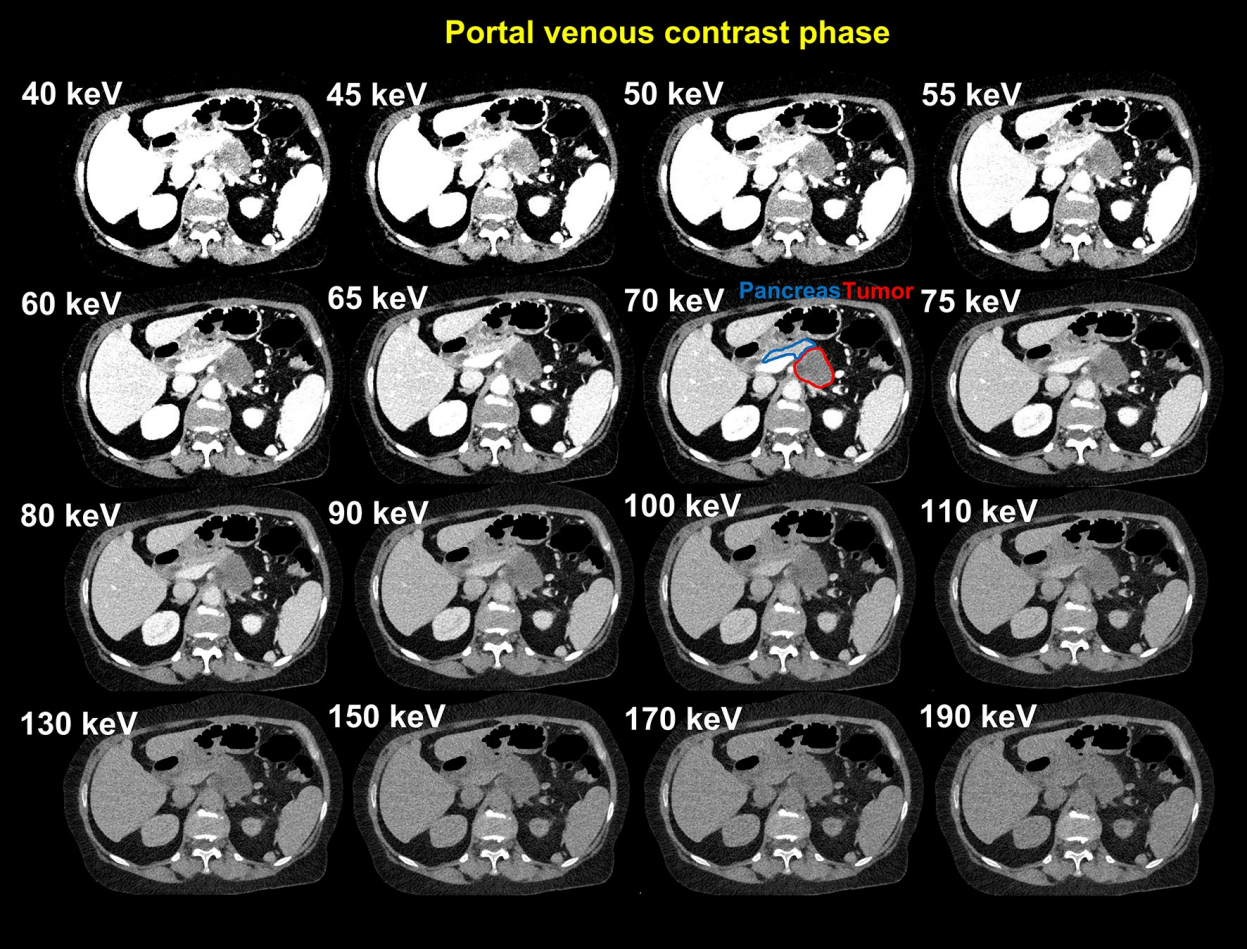
VMI reconstructions in the portal venous contrast phase in a patient with PDAC in the body/tail of the pancreas. *PDAC* pancreatic ductal adenocarcinoma

Figure 4 shows VMI reconstructions of a patient with PDAC in the body/tail in portal venous contrast phase. VMI reconstructions in arterial contrast phase are shown in Supplemental Fig. 1.

### Contrast phase

**Fig. 5:**
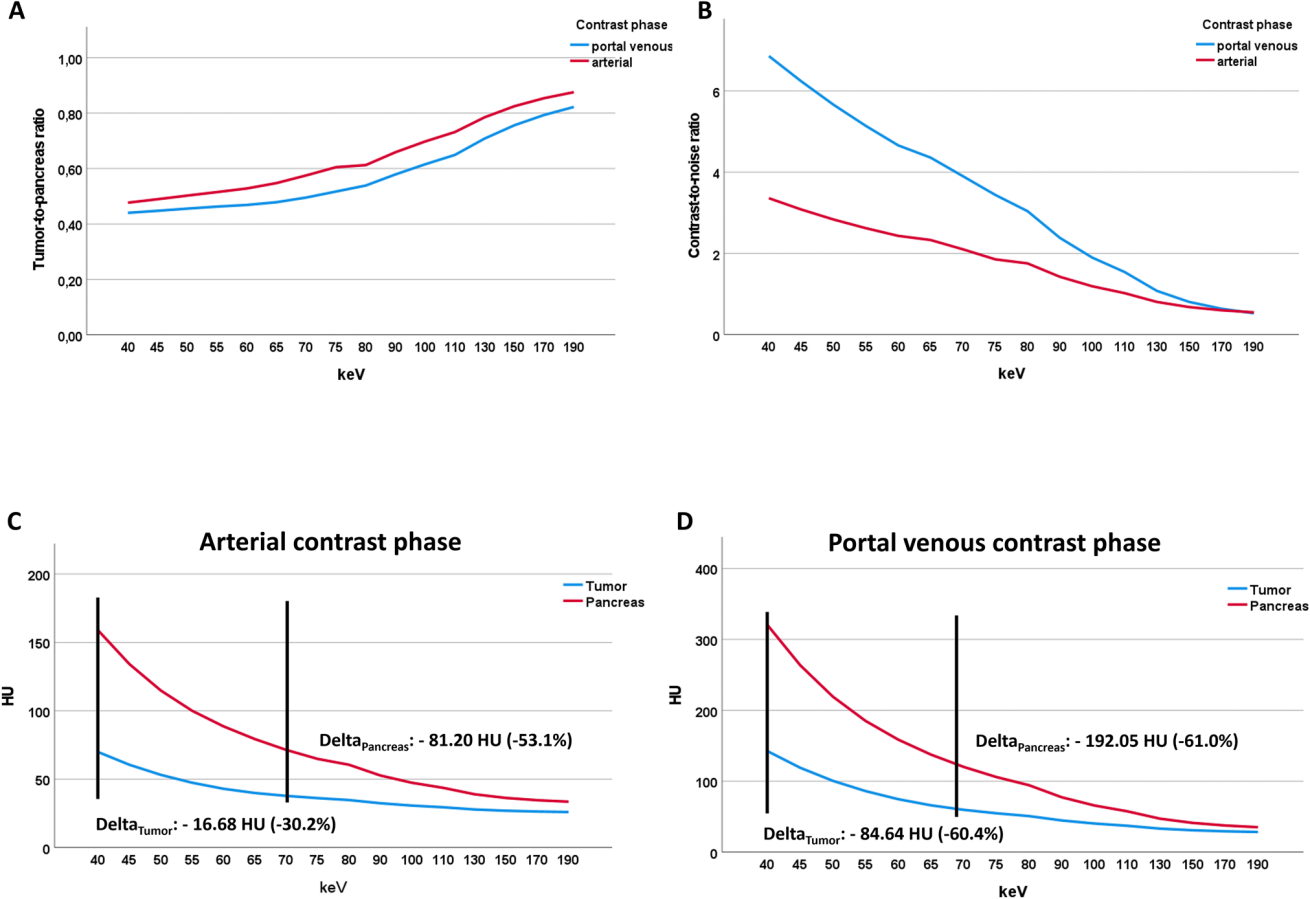
Tumor-to-pancreas ratio (TPR) (**A**) and contrast-to-noise ratio (CNR) (**B**) in comparison between arterial and portal venous contrast phase on the photon-counting detector CT. CT-values of tumor tissue and pancreatic tissue at different keV levels in arterial (**C**) and portal venous contrast phase (**D**) on the photon-counting detector CT

No significant differences in TPR were observed between arterial and portal venous contrast phases (Table 4). For all keV VMI reconstructions ≤ 110 keV, CNR was significantly higher in the portal venous contrast phase compared to the arterial contrast phase (e.g., 40 keV: 6.61 [4.39; 9.51] vs. 4.10 [0.94; 4.63], P = 0.016) (Table 4, Fig. 5). The difference in CNR between the arterial and portal venous phases is striking (Fig. 5B), reflecting the high tumor conspicuity at lower keV-levels in portal venous contrast phase compared to arterial contrast phase.

**Table 4:** Tumor-to-pancreas ratio and contrast-to-noise ratio at different contrast phases on a PCD-CT

keV	Arterial phase	Portal venous phase	P value
Tumor-to-pancreas ratio			
40	0.40 (0.26 to 0.79)	0.37 (0.20 to 0.62)	1.000
45	0.41 (0.27 to 0.82)	0.39 (0.21 to 0.65)	1.000
50	0.43 (0.28 to 0.85)	0.40 (0.22 to 0.65)	1.000
55	0.46 (0.29 to 0.87)	0.44 (0.23 to 0.65)	1.000
60	0.49 (0.30 to 0.88)	0.47 (0.25 to 0.65)	1.000
65	0.53 (0.30 to 0.86)	0.50 (0.26 to 0.66)	1.000
70	0.56 (0.32 to 0.85)	0.52 (0.28 to 0.68)	1.000
75	0.60 (0.34 to 0.89)	0.54 (0.30 to 0.71)	1.000
80	0.63 (0.35 to 0.84)	0.56 (0.30 to 0.73)	1.000
90	0.70 (0.37 to 0.90)	0.60 (0.30 to 0.78)	1.000
100	0.74 (0.38 to 0.95)	0.65 (0.34 to 0.81)	1.000
110	0.77 (0.38 to 1.00)	0.70 (0.36 to 0.84)	1.000
130	0.80 (0.40 to 1.09)	0.73 (0.41 to 0.92)	1.000
150	0.81 (0.40 to 1.12)	0.77 (0.44 to 0.98)	1.000
170	0.83 (0.40 to 1.15)	0.82 (0.46 to 1.01)	1.000
190	0.84 (0.41 to 1.19)	0.85 (0.48 to 1.05)	1.000
Contrast-to-noise ratio			
40	4.10 (0.94 to 4.63)	6.61 (4.39 to 9.51)	*0.016*
45	3.74 (0.77 to 4.19)	6.17 (3.90 to 8.64)	*0.016*
50	3.41 (0.67 to 3.93)	5.62 (3.47 to 7.84)	*0.016*
55	3.12 (0.62 to 3.76)	5.11 (3.08 to 7.13)	*0.016*
60	2.89 (0.50 to 3.58)	4.43 (2.80 to 6.47)	*0.016*
65	2.73 (0.44 to 3.67)	4.17 (2.70 to 5.95)	*0.016*
70	2.46 (0.44 to 3.36)	3.70 (2.39 to 5.31)	*0.016*
75	2.20 (0.39 to 3.05)	3.28 (2.06 to 4.73)	*0.016*
80	1.91 (0.87 to 2.75)	2.87 (1.82 to 4.22)	*0.016*
90	1.32 (0.49 to 2.32)	2.08 (1.44 to 3.31)	*0.016*
100	1.14 (0.23 to 2.09)	1.65 (1.14 to 2.69)	*0.016*
110	1.06 (0.00 to 1.91)	1.39 (0.82 to 2.23)	*0.048*
130	0.83 (− 0.28 to 1.72)	1.00 (0.37 to 1.74)	1.000
150	0.66 (− 0.39 to 1.61)	0.69 (0.08 to 1.45)	1.000
170	0.61 (− 0.49 to 1.53)	0.52 (− 0.05 to 1.28)	1.000
190	0.57 (− 0.55 to 1.48)	0.40 (− 0.15 to 1.21)	1.000

Data shown as median (interquartile range), P value shown after Bonferroni-correction

P value < 0.05 shown in italics

In portal venous contrast phase, both tissues showed a comparable decrease of CT-values with increasing keV levels (Table 5, Fig. 5D). However in arterial phase, decrease of CT-values was remarkably lower in tumor tissue compared to pancreatic tissue (Delta_70–40 keV_Tumor_: − 16.68 HU (− 30.2%) vs. Delta_70–40 keV_Pancreas_: − 81.20 HU (− 53.1%), Table 5, Fig. 5C).

**Table 5:** Median CT-values of tumor and pancreatic tissue at different keV levels

keV	Arterial phase		Portal venous phase	
	Tumor	Pancreas	Tumor	Pancreas
40	55.15 (37.60–85.91)	152.96 (95.88–181.73)	140.13 (60.96–211.03)	315.02 (278.90–374.15)
45	49.99 (35.83–74.88)	130.68 (82.10–153.35)	116.88 (52.67–177.06)	261.69 (230.63–305.02)
50	45.16 (33.52–66.26)	113.27 (71.44–131.12)	98.22 (46.23–148.92)	218.83 (192.80–251.16)
55	41.59 (30.36–58.17)	100.54 (62.90–113.98)	78.35 (41.42–128.04)	187.12 (162.70–210.82)
60	40.46 (26.97–54.36)	89.76 (56.25–101.39)	64.74 (37.80–110.75)	162.64 (137.23–178.59)
65	39.63 (24.54–51.50)	78.66 (51.38–91.60)	57.30 (34.86–97.45)	142.21 (118.33–154.32)
70	38.47 (23.40–49.94)	71.76 (47.83–83.23)	55.49 (32.26–87.46)	122.97 (103.35–136.20)
75	37.34 (22.94–47.71)	64.96 (45.14–75.87)	52.77 (30.04–79.97)	107.76 (92.03–119.91)
80	36.42 (22.23–45.78)	60.69 (49.48–69.82)	48.87 (27.52–74.08)	96.79 (81.72–108.93)
90	34.90 (20.51–42.82)	53.33 (42.04–60.89)	43.08 (23.43–65.13)	79.18 (67.16–88.02)
100	33.53 (19.17–40.92)	47.58 (37.86–56.07)	40.04 (21.69–57.98)	67.69 (57.16–74.21)
110	32.52 (18.09–39.87)	44.15 (36.96–52.71)	37.77 (20.77–51.64)	59.24 (50.40–64.81)
130	31.12 (16.71–38.70)	40.06 (33.22–46.32)	34.94 (19.79–47.90)	48.57 (42.86–53.23)
150	30.30 (15.90–38.01)	37.10 (30.55–41.15)	32.42 (19.22–45.26)	42.35 (35.59–47.47)
170	29.96 (15.37–37.58)	36.08 (28.84–39.22)	30.45 (18.90–42.45)	38.37 (31.74–43.73)
190	29.72 (15.06–37.26)	35.26 (27.75–38.28)	29.36 (18.79–40.58)	35.98 (29.40–41.56)

Data shown as median (interquartile range)

### Body Mass Index

In a subgroup analysis, patients were divided according to BMI using a median split (BMI = 22.5 kg/m^2^).

On the PCD-CT, TPR was significantly better in patients with a BMI < 22.5 kg/m^2^ in arterial phase, with however no significant differences in portal venous phase. In contrary, on the EID-CT, TPR was significantly better in patients with a BMI ≥ 22.5 kg/m^2^ (P < 0.001) in both phases. CNR was significantly higher in patients with lower BMI on the PCD-CT in arterial phase at higher keV reconstructions, whereas CNR was higher for patients with higher BMI on the EID-CT in arterial phase (Supplemental Table 2).

### Qualitative image analysis

**Fig. 6:**
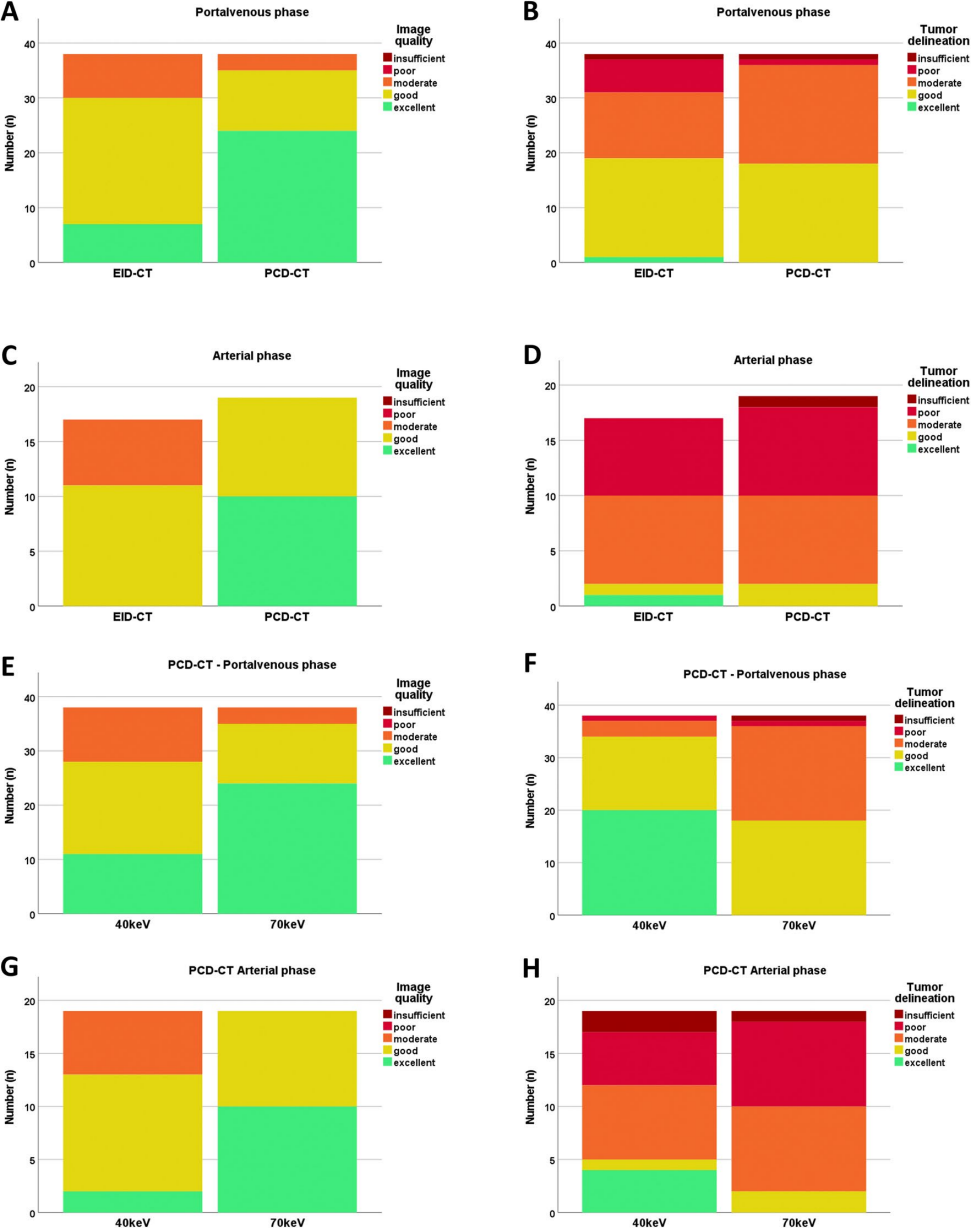
Subjective image analysis. Rating of two board-certified radiologists for image quality and tumor delineation in comparison between EID-CT and PCD-CT in portal venous phase (**A**, **B**) and arterial phase (**C**, **D**). Comparison between 40 and 70 keV on a PCD-CT for image quality and tumor delineation in portal venous phase (**E**, **F**) and arterial phase (**G**, **H**)

Comparison between EID-CT and PCD-CT showed improved image quality on a PCD-CT at both contrast phases. However, tumor delineation was not better on a PCD-CT compared to EID-CT at 70 keV reconstructions. Considering only PCD-CT scans, tumor delineation was improved at 40 keV compared to 70 keV in both, arterial and portal venous contrast phase, despite subjective lower image quality (Fig. 6).

## Discussion

PCD-CT showed significantly improved tumor conspicuity (reflected by low TPR and high CNR) at lower keV-levels (≤ 70 keV) in arterial and portal venous contrast phases. Compared to EID-CT, tumor delineation on PCD-CT is superior only in the portal venous phase, not in the arterial phase. Tumor tissue showed a slower decrease of CT-values with increasing keV levels compared to pancreatic tissue in arterial phase, which may also be helpful for the diagnosis. Subjective image analysis showed improved tumor delineation at lower keV levels compared to 70 keV in both, arterial and portal venous phase.

Improved delineation of PDAC at lower keV-levels has previously been shown on dual-energy CT [16, 19–22, 30], but to the best of our knowledge not on a PCD-CT. Best tumor conspicuity as well as best objective and subjective image quality was demonstrated at 40 keV [16]. At lower x-ray energies the iodine signal increases resulting in an improved contrast between enhancing tissue (e.g., pancreas) and tissue with reduced contrast enhancement (e.g., PDAC). With increasing availability of PCD-CT, spectral data acquisition is routinely performed on every scan, without increasing radiation dose or dedicated protocols. Previous studies have also highlighted the benefits of low-keV reconstructions in abdominal imaging, demonstrating superior CNR and good subjective image quality [27, 31]. Improved delineation of hypovascular liver metastases as well as better objective and subjective image quality in oncological imaging have been shown on a novel PCD-CT at lower keV levels (e.g. 40 keV) [15, 32].

Our study shows higher image noise at lower keV levels. Similar results have been published previously [15, 27, 33]. However, it has been shown for abdominal CT that subjective image quality was higher at lower keV levels despite higher image noise [27]. In other studies investigating VMI for other purposes (e.g., vessel delineation), lower keV levels were also preferred by radiologists [32, 34–36]. Therefore, contrast-to-noise ratio may be considered superior to image noise in the evaluation of contrast-enhanced CT scans.

Two phase acquisition (arterial phase and portal venous phase) has been shown to provide the best delineation of PDAC and is therefore routinely performed in patients with suspected pancreatic tumors [37, 38]. This study shows superior CNR at lower keV levels compared to higher keV levels for both—arterial and venous contrast phases. However, especially at lower keV levels (< 70 keV), CNR was remarkably higher in portal venous contrast phase compared to arterial contrast phase. In arterial contrast phase, we observed a higher difference in CT-values (between 40 and 70 keV) in pancreatic tissue compared to tumor tissue. These findings highlight the importance of both, arterial and portal venous contrast phases in the imaging of PDAC. Further studies with larger patient cohorts are needed to evaluate the utility of these findings in clinical routine.

When looking at the impact of BMI, we observed that the tumor conspicuity was improved (lower TPR) in patients with higher BMI on the EID-CT in both arterial and portal venous phase. On the PCD-CT on the other hand, there were no significant differences between patients with lower and higher BMI in portal venous phase. This may be due to better image quality and lower noise in PCD-CT scans of patients with higher BMI which is most likely caused by the adequate weighting of low-energy photons on PCD-CT. These findings are in line with previous studies on the comparison of abdominal EID- and PCD (low dose) CT where scans of patients with higher BMI showed no significant increase in noise with stable SNR compared with low-BMI scans [15, 39].

In this study, two board-certified radiologists independently reviewed the CT scans and rated them on a 5-point Likert scale regarding both, image quality and tumor delineation. Similar results compared to the quantitative analyses were shown. At 70 keV, no better tumor delineation was reported on a PCD-CT compared to the EID-CT. However, at 40 keV radiologists recorded improved tumor delineation compared to 70 keV, both in arterial and portal venous phase. Interestingly, the better subjective tumor delineation was also recorded despite lower subjective image quality at lower keV levels, which might be due to higher image noise.

This study has several limitations. First, the retrospective single-center study design is a major limitation. CT scans were performed during clinical routine; therefore, there are differences in image acquisition protocols between PCD-CT and EID-CT, and not all patients received combined arterial and portal venous phase imaging. Second, the small number of patients is a limitation. However, PDAC is a rare disease. Third, we did not match patients for BMI, age, and gender because of the small number of available patients with an initial diagnosis of PDAC. Therefore, there might be a bias in image noise and CNR due to differences, especially in BMI. However, in the overall patient cohort, BMI and also other demographic parameters were not significantly different. Further, we analyzed different patients that received imaging on different CT scanners and did not perform a head-to-head comparison which might introduce a further bias. Fourth, as the EID-CT does not contain a dual-energy mode, it was not possible to perform direct comparisons between VMI’s reconstructed on DECT and PCD-CT. Future studies might consider this to analyze the differences between both VMI’s.

## Conclusion

Implementation of VMI with low keV levels (e.g. 40 keV) for both—arterial and portal venous phase—in clinical routine may improve delineation of pancreatic ductal adenocarcinoma in patients with suspected pancreatic cancer.

### Supplementary Information

Below is the link to the electronic supplementary material.

Supplementary file1 (GIF 4882 kb)

Supplementary file2 (DOCX 17 kb)

Supplementary file3 (DOCX 25 kb)

## Abbreviations

CNR: Contrast-to-noise ratio
CT: Computed tomography
DECT: Dual-energy CT
EID-CT: Energy integrating detector CT
keV: Kiloelectronvolt
PCD-CT: Photon counting detector CT
PDAC: Pancreatic ductal adenocarcinoma
SNR: Signal-to-noise ratio
TPR: Tumor-to-pancreas ratio
VMI: Virtual monoenergetic images

## References

[1] Arnold M, Rutherford MJ, Bardot A, Ferlay J, Andersson TM-L, Myklebust TÅ, et al. Progress in cancer survival, mortality, and incidence in seven high-income countries 1995-2014 (ICBP SURVMARK-2): a population-based study. Lancet Oncol 2019;20:1493–505. 10.1016/S1470-2045(19)30456-5. 10.1016/S1470-2045(19)30456-5 PMC6838671 31521509

[2] Bray F, Ferlay J, Soerjomataram I, Siegel RL, Torre LA, Jemal A (2018). Global cancer statistics 2018: GLOBOCAN estimates of incidence and mortality worldwide for 36 cancers in 185 countries. CA Cancer J Clin.

[3] Allemani C, Matsuda T, di Carlo V, Harewood R, Matz M, Nikšić M (2018). Global surveillance of trends in cancer survival 2000–14 (CONCORD-3): analysis of individual records for 37 513 025 patients diagnosed with one of 18 cancers from 322 population-based registries in 71 countries. Lancet.

[4] Rahib L, Smith BD, Aizenberg R, Rosenzweig AB, Fleshman JM, Matrisian LM (2014). Projecting cancer incidence and deaths to 2030: the unexpected burden of thyroid, liver, and pancreas cancers in the United States. Cancer Res.

[5] Pereira SP, Oldfield L, Ney A, Hart PA, Keane MG, Pandol SJ (2020). Early detection of pancreatic cancer. Lancet Gastroenterol Hepatol.

[6] Singhi AD, Koay EJ, Chari ST, Maitra A (2019). Early Detection of Pancreatic Cancer: Opportunities and Challenges. Gastroenterology.

[7] Canto MI, Hruban RH, Fishman EK, Kamel IR, Schulick R, Zhang Z, et al. Frequent detection of pancreatic lesions in asymptomatic high-risk individuals. Gastroenterology 2012;142:796–804; quiz e14-5. 10.1053/j.gastro.2012.01.005. 10.1053/j.gastro.2012.01.005 PMC3321068 22245846

[8] Sheridan MB, Ward J, Guthrie JA, Spencer JA, Craven CM, Wilson D (1999). Dynamic contrast-enhanced MR imaging and dual-phase helical CT in the preoperative assessment of suspected pancreatic cancer: a comparative study with receiver operating characteristic analysis. AJR Am J Roentgenol.

[9] Palazzo L, Roseau G, Gayet B, Vilgrain V, Belghiti J, Fékéte F, et al. Endoscopic ultrasonography in the diagnosis and staging of pancreatic adenocarcinoma. Results of a prospective study with comparison to ultrasonography and CT scan. Endoscopy 1993;25:143–50. 10.1055/s-2007-1010273. 10.1055/s-2007-1010273 8491130

[10] Kauhanen SP, Komar G, Seppänen MP, Dean KI, Minn HR, Kajander SA (2009). A prospective diagnostic accuracy study of 18F-fluorodeoxyglucose positron emission tomography/computed tomography, multidetector row computed tomography, and magnetic resonance imaging in primary diagnosis and staging of pancreatic cancer. Ann Surg.

[11] Sahani DV, Shah ZK, Catalano OA, Boland GW, Brugge WR (2008). Radiology of pancreatic adenocarcinoma: current status of imaging. J Gastroenterol Hepatol.

[12] Legmann P, Vignaux O, Dousset B, Baraza AJ, Palazzo L, Dumontier I (1998). Pancreatic tumors: comparison of dual-phase helical CT and endoscopic sonography. AJR Am J Roentgenol.

[13] Fletcher JG, Wiersema MJ, Farrell MA, Fidler JL, Burgart LJ, Koyama T (2003). Pancreatic malignancy: value of arterial, pancreatic, and hepatic phase imaging with multi-detector row CT. Radiology.

[14] Lenga L, Czwikla R, Wichmann JL, Leithner D, Albrecht MH, Booz C (2018). Dual-energy CT in patients with colorectal cancer: Improved assessment of hypoattenuating liver metastases using noise-optimized virtual monoenergetic imaging. Eur J Radiol.

[15] Bette S, Decker JA, Braun FM, Becker J, Haerting M, Haeckel T, et al. Optimal Conspicuity of Liver Metastases in Virtual Monochromatic Imaging Reconstructions on a Novel Photon-Counting Detector CT-Effect of keV Settings and BMI. Diagnostics (Basel) 2022;12. 10.3390/diagnostics12051231. 10.3390/diagnostics12051231 PMC9140684 35626387

[16] Liang H, Zhou Y, Zheng Q, Yan G, Liao H, Du S (2022). Dual-energy CT with virtual monoenergetic images and iodine maps improves tumor conspicuity in patients with pancreatic ductal adenocarcinoma. Insights Imaging.

[17] D’Angelo T, Cicero G, Mazziotti S, Ascenti G, Albrecht MH, Martin SS (2019). Dual energy computed tomography virtual monoenergetic imaging: technique and clinical applications. Br J Radiol.

[18] Hanson GJ, Michalak GJ, Childs R, McCollough B, Kurup AN, Hough DM (2018). Low kV versus dual-energy virtual monoenergetic CT imaging for proven liver lesions: what are the advantages and trade-offs in conspicuity and image quality?. A pilot study. Abdom Radiol (NY).

[19] Nagayama Y, Tanoue S, Inoue T, Oda S, Nakaura T, Utsunomiya D (2020). Dual-layer spectral CT improves image quality of multiphasic pancreas CT in patients with pancreatic ductal adenocarcinoma. Eur Radiol.

[20] Beer L, Toepker M, Ba-Ssalamah A, Schestak C, Dutschke A, Schindl M, et al. Objective and subjective comparison of virtual monoenergetic vs. polychromatic images in patients with pancreatic ductal adenocarcinoma. Eur Radiol 2019;29:3617–25. 10.1007/s00330-019-06116-9. 10.1007/s00330-019-06116-9 PMC6554239 30888484

[21] Noda Y, Goshima S, Kaga T, Ando T, Miyoshi T, Kawai N (2020). Virtual monochromatic image at lower energy level for assessing pancreatic ductal adenocarcinoma in fast kV-switching dual-energy CT. Clin Radiol.

[22] Noda Y, Takai Y, Asano M, Yamada N, Seko T, Kawai N, et al. Comparison of image quality and pancreatic ductal adenocarcinoma conspicuity between the low-kVp and dual-energy CT reconstructed with deep-learning image reconstruction algorithm. Eur J Radiol 2023;159:110685. 10.1016/j.ejrad.2022.110685. 10.1016/j.ejrad.2022.110685 36603479

[23] Fujisaki Y, Fukukura Y, Kumagae Y, Ejima F, Yamagishi R, Nakamura S, et al. Value of Dual-Energy Computed Tomography for Detecting Small Pancreatic Ductal Adenocarcinoma. Pancreas n.d.;51:1352–8. 10.1097/MPA.0000000000002207. 10.1097/MPA.0000000000002207 37099778

[24] Willemink MJ, Persson M, Pourmorteza A, Pelc NJ, Fleischmann D (2018). Photon-counting CT: Technical Principles and Clinical Prospects. Radiology.

[25] Flohr T, Petersilka M, Henning A, Ulzheimer S, Ferda J, Schmidt B (2020). Photon-counting CT review. Phys Med.

[26] Bette SJ, Braun FM, Haerting M, Decker JA, Luitjens JH, Scheurig-Muenkler C (2021). Visualization of bone details in a novel photon-counting dual-source CT scanner-comparison with energy-integrating CT. Eur Radiol.

[27] Higashigaito K, Euler A, Eberhard M, Flohr TG, Schmidt B, Alkadhi H (2021). Contrast-Enhanced Abdominal CT with Clinical Photon-Counting Detector CT: Assessment of Image Quality and Comparison with Energy-Integrating Detector CT. Acad Radiol.

[28] Schindelin J, Arganda-Carreras I, Frise E, Kaynig V, Longair M, Pietzsch T (2012). Fiji: an open-source platform for biological-image analysis. Nat Methods.

[29] Nagayama Y, Iyama A, Oda S, Taguchi N, Nakaura T, Utsunomiya D (2019). Dual-layer dual-energy computed tomography for the assessment of hypovascular hepatic metastases: impact of closing k-edge on image quality and lesion detectability. Eur Radiol.

[30] Frellesen C, Fessler F, Hardie AD, Wichmann JL, De Cecco CN, Schoepf UJ (2015). Dual-energy CT of the pancreas: improved carcinoma-to-pancreas contrast with a noise-optimized monoenergetic reconstruction algorithm. Eur J Radiol.

[31] Booij R, van der Werf NR, Dijkshoorn ML, van der Lugt A, van Straten M. Assessment of Iodine Contrast-To-Noise Ratio in Virtual Monoenergetic Images Reconstructed from Dual-Source Energy-Integrating CT and Photon-Counting CT Data. Diagnostics (Basel) 2022;12. 10.3390/diagnostics12061467. 10.3390/diagnostics12061467 PMC9222007 35741277

[32] Graafen D, Müller L, Halfmann M, Düber C, Hahn F, Yang Y, et al. Photon-counting detector CT improves quality of arterial phase abdominal scans: A head-to-head comparison with energy-integrating CT. Eur J Radiol 2022;156:110514. 10.1016/j.ejrad.2022.110514. 10.1016/j.ejrad.2022.110514 36108479

[33] Dillinger D, Overhoff D, Booz C, Kaatsch HL, Piechotka J, Hagen A, et al. Impact of CT Photon-Counting Virtual Monoenergetic Imaging on Visualization of Abdominal Arterial Vessels. Diagnostics (Basel) 2023;13. 10.3390/diagnostics13050938. 10.3390/diagnostics13050938 PMC10000913 36900082

[34] Dunning CAS, Rajendran K, Inoue A, Rajiah P, Weber N, Fletcher JG (2023). Optimal Virtual Monoenergetic Photon Energy (keV) for Photon-Counting-Detector Computed Tomography Angiography. J Comput Assist Tomogr.

[35] Rippel K, Decker JA, Wudy R, Trzaska T, Haerting M, Kroencke TJ, et al. Evaluation of run-off computed tomography angiography on a first-generation photon-counting detector CT scanner - Comparison with low-kVp energy-integrating CT. Eur J Radiol 2023;158:110645. 10.1016/j.ejrad.2022.110645. 10.1016/j.ejrad.2022.110645 36525704

[36] Euler A, Higashigaito K, Mergen V, Sartoretti T, Zanini B, Schmidt B (2022). High-Pitch Photon-Counting Detector Computed Tomography Angiography of the Aorta: Intraindividual Comparison to Energy-Integrating Detector Computed Tomography at Equal Radiation Dose. Invest Radiol.

[37] Lu DS, Vedantham S, Krasny RM, Kadell B, Berger WL, Reber HA (1996). Two-phase helical CT for pancreatic tumors: pancreatic versus hepatic phase enhancement of tumor, pancreas, and vascular structures. Radiology.

[38] Boland GW, O’Malley ME, Saez M, Fernandez-del-Castillo C, Warshaw AL, Mueller PR (1999). Pancreatic-phase versus portal vein-phase helical CT of the pancreas: optimal temporal window for evaluation of pancreatic adenocarcinoma. AJR Am J Roentgenol.

[39] Decker JA, Bette S, Lubina N, Rippel K, Braun F, Risch F, et al. Low-dose CT of the abdomen: Initial experience on a novel photon-counting detector CT and comparison with energy-integrating detector CT. Eur J Radiol 2022;148:110181. 10.1016/j.ejrad.2022.110181. 10.1016/j.ejrad.2022.110181 35121331

