# Supplementary figures and images for: Optimal conspicuity of pancreatic ductal adenocarcinoma in virtual monochromatic imaging reconstructions on a photon-counting detector CT: comparison to conventional MDCT
Source: Abdom Radiol (NY). 2023 Oct 5;49(1):103–16. doi: 10.1007/s00261-023-04042-5 (PMC10789688; doi:10.1007/s00261-023-04042-5)

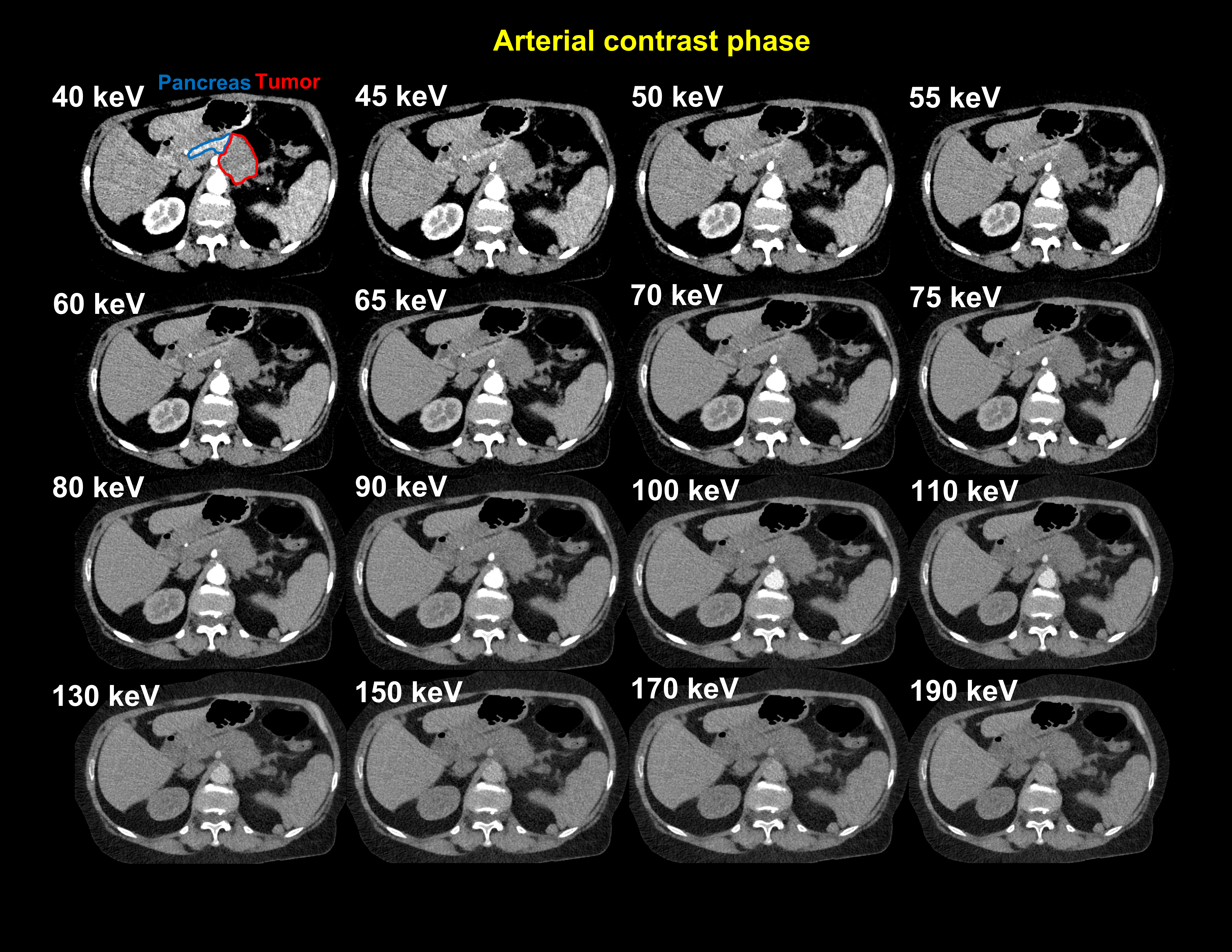

Supplement: Supplementary file 1 — Supplementary file1 (GIF 4882 kb) [file 261_2023_4042_MOESM1_ESM.gif]
