# Supplementary material for: Optimal conspicuity of pancreatic ductal adenocarcinoma in virtual monochromatic imaging reconstructions on a photon-counting detector CT: comparison to conventional MDCT
Source: Abdom Radiol (NY). 2023 Oct 5;49(1):103–16. doi: 10.1007/s00261-023-04042-5 (PMC10789688; doi:10.1007/s00261-023-04042-5)
Supplement: Supplementary file 2 — Supplementary file2 (DOCX 17 kb) [file 261_2023_4042_MOESM2_ESM.docx]

| **Supplemental Table 1 Median image noise at different keV levels and contrast phases** | | | | | | |
| --- | --- | --- | --- | --- | --- | --- |
|  | **Arterial phase** | |  | **Portal venous phase** | |  |
| **keV** | **PCD-CT** | **EID-CT** | **P-Value** | **PCD-CT** | **EID-CT** | **P-Value** |
| 40 | 28.7 (24.5-33.8) | 14.4 (12.4-17.3) | *0.016* | 31.8 (25.9-36.8) | 17.3 (13.8-20.8) | *0.016* |
| 45 | 26.1 (22.0-30.5) |  | *0.016* | 28.2 (22.9-32.3) |  | *0.016* |
| 50 | 23.6 (19.9-27.3) |  | *0.016* | 25.3 (20.9-28.6) |  | *0.016* |
| 55 | 21.5 (17.9-24.9) |  | *0.016* | 22.8 (19.2-26.0) |  | *0.016* |
| 60 | 19.6 (16.6-22.7) |  | *0.016* | 20.9 (18.0-24.0) |  | *0.016* |
| 65 | 17.3 (14.6-20.3) |  | *0.016* | 18.4 (15.9-21.3) |  | 0.096 |
| 70 | 16.3 (13.7-19.0) |  | *0.016* | 17.1 (14.7-19.8) |  | 1.000 |
| 75 | 16.0 (13.6-18.2) |  | *0.032* | 16.4 (14.2-18.6) |  | *0.032* |
| 80 | 15.6 (13.4-17.7) |  | 0.464 | 15.8 (13.7-18.2) |  | *0.016* |
| 90 | 15.3 (13.1-17.1) |  | 1.000 | 15.3 (13.3-17.6) |  | *0.016* |
| 100 | 15.0 (12.9-16.9) |  | 1.000 | 15.1 (12.9-17.2) |  | *0.016* |
| 110 | 14.9 (12.8-16.8) |  | 1.000 | 15.0 (12.7-17.1) |  | *0.016* |
| 130 | 14.6 (12.7-16.7) |  | 1.000 | 14.9 (12.7-16.9) |  | *0.016* |
| 150 | 14.6 (12.7-16.7) |  | 1.000 | 14.9 (12.7-16.8) |  | *0.016* |
| 170 | 14.5 (12.7-16.7) |  | 1.000 | 14.9 (12.6-16.8) |  | *0.016* |
| 190 | 14.5 (12.6-16.6) |  | 1.000 | 14.9 (12.6-16.8) |  | *0.016* |

Image noise measured in all ROIs. Data shown as median (interquartile range). P-Value shown after Bonferroni-correction. P-Value < 0.05 shown in *italics*
