# Supplementary material for: Optimal conspicuity of pancreatic ductal adenocarcinoma in virtual monochromatic imaging reconstructions on a photon-counting detector CT: comparison to conventional MDCT
Source: Abdom Radiol (NY). 2023 Oct 5;49(1):103–16. doi: 10.1007/s00261-023-04042-5 (PMC10789688; doi:10.1007/s00261-023-04042-5)
Supplement: Supplementary file 3 — Supplementary file3 (DOCX 25 kb) [file 261_2023_4042_MOESM3_ESM.docx]

| **Supplemental Table 2 Tumor-to-pancreas ratio and Contrast-to-noise ratio at different keV levels, divided into groups according to BMI** | | | | | | | | | | | | | |
| --- | --- | --- | --- | --- | --- | --- | --- | --- | --- | --- | --- | --- | --- |
|  | **PCD-CT** | | | | | | **EID-CT** | | | | | | |
|  | **Arterial phase** | | | | | | | | | | | | |
| **keV** | **Tumor-to-pancreas ratio** | | **P-Value** | **Contrast-to-noise ratio** | | **P-Value** | **Tumor-to-pancreas ratio** | | **P-Value** | **Contrast-to-noise ratio** | | **P-Value** |  |
|  | ***BMI < 22.5*** | ***BMI ≥ 22.5*** |  | ***BMI < 22.5*** | ***BMI ≥ 22.5*** |  | ***BMI < 22.5*** | ***BMI ≥ 22.5*** |  | ***BMI < 22.5*** | ***BMI ≥ 22.5*** |  |  |
| 40 | 0.36 (0.26-0.48) | 0.67 0.32-0.82 | 0.144 | 4.17 3.51-5.03 | 3.89 0.89-4.53 | 1.000 | 0.63 (0.51-0.81) | 0.39 (0.32-0.52) | *<0.001* | 2.11 (1.06-2.84) | 3.34 (1.95-5.05) | *<0.001* |  |
| 45 | 0.36 (0.26-0.49) | 0.67 0.35-0.86 | 0.080 | 3.89 3.27-4.64 | 3.57 0.72-4.09 | 1.000 |  |  |  |  |  |  |  |
| 50 | 0.35 (0.27-0.52) | 0.68 0.38-0.89 | *0.032* | 3.64 3.01-4.28 | 3.29 0.53-3.74 | 1.000 |  |  |  |  |  |  |  |
| 55 | 0.34 (0.28-0.53) | 0.69 0.41-0.93 | *0.016* | 3.42 2.79-3.87 | 2.99 0.33-3.50 | 0.704 |  |  |  |  |  |  |  |
| 60 | 0.34 (0.29-0.54) | 0.71 0.44-0.96 | *0.016* | 3.20 2.59-3.64 | 2.61 0.17 3.50 | 0.025 |  |  |  |  |  |  |  |
| 65 | 0.35 (0.30-0.56) | 0.73 0.49-0.99 | *0.016* | 3.02 2.46-3-68 | 2.27 0.02 3.55 | 0.128 |  |  |  |  |  |  |  |
| 70 | 0.36 (0.31-0.58) | 0.77 0.54-1.00 | *0.016* | 2.82 2.32-3.54 | 1.82 0.00-2.86 | *0.016* |  |  |  |  |  |  |  |
| 75 | 0.36 (0.33-0.59) | 0.81 0.60-1.00 | *0.016* | 2.64 2.20-3.20 | 1.37 0.00-2.15 | *0.016* |  |  |  |  |  |  |  |
| 80 | 0.38 (0.34-0.62) | 0.82 0.65-0.87 | *0.016* | 2.48 2.11-2.87 | 1.08 0.69-1.65 | *0.016* |  |  |  |  |  |  |  |
| 90 | 0.41 (0.36-0.66) | 0.86 0.74-0.95 | *0.016* | 2.15 1.68-2.40 | 0.64 0.26-0.97 | *0.016* |  |  |  |  |  |  |  |
| 100 | 0.43 (0.36-0.69) | 0.92 0.82-1.00 | *0.016* | 1.95 1.36-2.20 | 0.35 0.00-0.63 | *0.016* |  |  |  |  |  |  |  |
| 110 | 0.44 (0.36-0.73) | 0.97 0.88-1.00 | *0.016* | 1.77 1.14-2.07 | 0.11 0.00-0.38 | *0.016* |  |  |  |  |  |  |  |
| 130 | 0.45 (0.37-0.78) | 1.00 0.96-1.00 | *0.016* | 1.52 0.97-1.92 | 0.00 0.00-0.16 | *0.016* |  |  |  |  |  |  |  |
| 150 | 0.47 (0.37-0.79) | 1.00 0.98-1.00 | *0.016* | 1.37 0.78-1.83 | 0.00 0.00-0.04 | *0.016* |  |  |  |  |  |  |  |
| 170 | 0.48 (0.37-0.79) | 1.00 1.00-1.00 | *0.016* | 1.28 0.66-1.79 | 0.00 0.00-0.00 | *0.016* |  |  |  |  |  |  |  |
| 190 | 0.49 (0.37-0.79) | 1.00 1.00-1.00 | *0.016* | 1.22 0.61-1.75 | 0.00 0.00-0.00 | *0.016* |  |  |  |  |  |  |  |
|  | **Portal venous phase** | | | | | | | | | | | |  |
| **keV** | **Tumor-to-pancreas ratio** | | **P-Value** | **Contrast-to-noise ratio** | | **P-Value** | **Tumor-to-pancreas ratio** | | **P-Value** | **Contrast-to-noise ratio** | | **P-Value** |  |
|  | ***BMI < 22.5*** | ***BMI ≥ 22.5*** |  | ***BMI < 22.5*** | ***BMI ≥ 22.5*** |  | ***BMI < 22.5*** | ***BMI ≥ 22.5*** |  | ***BMI < 22.5*** | ***BMI ≥ 22.5*** |  |  |
| 40 | 0.29 (0.18-0.61) | 0.49 0.32-0.69 | 0.112 | 6.91 4.46-0.89 | 5.87 4.14-8.41 | 1.000 | 0.57 (0.48-0.64) | 0.40 (0.24-0.50) | *<0.001* | 3.33 (2.25-4.35) | 3.68 (2.63-4.29) | 0.197 |  |
| 45 | 0.30 (0.18-0.62) | 0.49 0.33-0.69 | 0.023 | 6.61 3.93-8.92 | 5.28 3.72-7.54 | 1.000 |  |  |  |  |  |  |  |
| 50 | 0.31 (0.17-0.63) | 0.50 0.33-0.70 | 0.112 | 6.10 3.50-8.10 | 4.74 3.34-6.76 | 1.000 |  |  |  |  |  |  |  |
| 55 | 0.32 (0.18-0.63) | 0.51 0.32-0.70 | 0.064 | 5.62 3.12-7.34 | 4.31 3.00-6.08 | 1.000 |  |  |  |  |  |  |  |
| 60 | 0.33 (0.19-0.63) | 0.52 0.32-0.70 | 0.064 | 5.19 2.88-6.56 | 3.98 2.64-5.61 | 0.720 |  |  |  |  |  |  |  |
| 65 | 0.34 (0.20-0.65) | 0.54 0.32-0.70 | 0.064 | 4.81 2.72-6.00 | 3.71 2.39-5.49 | 1.000 |  |  |  |  |  |  |  |
| 70 | 0.36 (0.21-0.66) | 0.56 0.32-0.72 | 0.080 | 4.29 2.44-5.33 | 3.37 2.15-5.17 | 1.000 |  |  |  |  |  |  |  |
| 75 | 0.38 (0.22-0.68) | 0.57 0.32-0.73 | 0.192 | 3.91 2.20-4.73 | 2.96 1.93-4.70 | 1.000 |  |  |  |  |  |  |  |
| 80 | 0.41 (0.23-0.71) | 0.59 0.34-0.75 | 0.416 | 3.61 1.96-4.26 | 3.58 1.71-4.16 | 1.000 |  |  |  |  |  |  |  |
| 90 | 0.46 (0.26-0.78) | 0.64 0.38-0.79 | 0.928 | 3.02 1.55-3.41 | 1.87 1.30-3.18 | 0.752 |  |  |  |  |  |  |  |
| 100 | 0.51 (0.29-0.80) | 0.69 0.44-0.82 | 0.048 | 2.34 1.27-2.95 | 1.37 1.01-2.49 | 0.203 |  |  |  |  |  |  |  |
| 110 | 0.56 (0.31-0.82) | 0.73 0.49-0.87 | 0.256 | 1.83 1.03-2.73 | 1.01 0.70-1.95 | 0.096 |  |  |  |  |  |  |  |
| 130 | 0.66 (0.34-0.86) | 0.81 0.54-0.96 | 0.128 | 1.28 0.68-2.16 | 0.68 0.15-1.39 | 0.016 |  |  |  |  |  |  |  |
| 150 | 0.72 (0.36-0.88) | 0.87 0.63-1.00 | 0.064 | 0.95 0.54-1.70 | 0.46 0.00-1.04 | 0.016 |  |  |  |  |  |  |  |
| 170 | 0.75 (0.36 0.89) | 0.89 0.65-1.00 | 0.032 | 0.81 0.35-1.50 | 0.34 0.00-0.81 | 0.016 |  |  |  |  |  |  |  |
| 190 | 0.78 (0.37-0.92) | 0.92 0.67-1.00 | 0.032 | 0.73 0.17-1.43 | 0.23 0.00-0.66 | 0.016 |  |  |  |  |  |  |  |

Data shown as median (interquartile range), P-Value < 0.05 shown in *Italics*
